# Evaluation of the cost of severe sepsis and septic shock in a private ICU in Brazil

**DOI:** 10.1186/cc14100

**Published:** 2015-03-16

**Authors:** M Borges Velasco, MA Leitão, MB Leitão, DM Dalcomune, LP Massete

**Affiliations:** 1 Hospital Meridional S.A., Vila Velha, Brazil

## Introduction

Sepsis is a high-prevalence disease in ICUs, associated with high mortality and high costs, mainly in developing countries. The aim of this study is to demonstrate the ICU costs, in a private hospital, in patients admitted with severe sepsis and septic shock.

## Methods

A retrospective, observational, single-center study of patients admitted from November 2013 to March 2014 with severe sepsis and septic shock. The records data were taken from the Software Epimed, MV System, and IBM SPSS Statistics 21. The classification was based on the Surviving Sepsis Campaign 2012. We included all 50 beds of an adult ICU, clinical and surgical. All patients older than 18 years with severe sepsis and septic shock were included. We evaluated the costs of patients during their ICU stay, and its relation to clinical presentation (severe sepsis and septic shock), antibiotic start time, permanence of ICU stay, and mortality. Only the first episode per patient was recorded.

## Results

From November 2013 to March 2014 were included 82 patients with criteria for severe sepsis and septic shock. The mean age of patients was 62.5 ± 21.8 years, divided equally between the genres. The overall mortality rate was 34.15%. The SAPS 3 was 56.43, with death probability set to Latin America 38.83%. Patients with severe sepsis had a mortality of 23.2% and those with septic shock had a mortality rate of 58%. The average total cost during ICU admission per patient was US$17,834 and the average daily cost was US$1,641. The daily cost in patients with severe sepsis and septic shock was US$1,263 and US$2,465 (*P *= 0.002), respectively, and in survivors and nonsurvivors was US$1,189 and US$2,512 (*P *= 0.001). The length of stay of patients in the ICU was 11.09 days, being 11.3 days in patients with severe sepsis and 10.7 days in patients with septic shock (*P *= 0.785). The beginning of the antibiotics in nonsurvivors was 73.7 minutes and in survivors was 64.7 minutes (*P *= 0.757), with the earliest onset in patients with septic shock than in patients with severe sepsis (38.5 vs. 81.5 minutes, *P *= 0.141).

## Conclusion

Severe sepsis and septic shock are conditions that consume large amounts of resources. Nonsurvivors had higher average spending than survivors. Patients admitted with septic shock had higher mortality than patients with severe sepsis with high mortality in relation to the prognostic indices adopted. The beginning of the antibiotics was longer in the nonsurvivors. We should adopt measures aimed at recognizing and earlier treatment of sepsis. If we improve our treatment, especially in septic shock, we will prevent deaths and decrement costs.

